# Commentary: Distribution of 5-HT_1F_ Receptors in Monkey Vestibular and Trigeminal Ganglion Cells

**DOI:** 10.3389/fneur.2016.00234

**Published:** 2016-12-20

**Authors:** Marcelo M. Valença

**Affiliations:** ^1^Neurosurgery and Neurology Unit, Hospital das Clínicas, Federal University of Pernambuco, Cidade Universitária, Recife, Pernambuco, Brazil

**Keywords:** serotonin, vestibular migraine, vomiting, receptor, pathophysiology, headache, 5-HT

Based on the assumption that a congruence exists between the expression of serotonin (5-hydroxytryptamine, 5-HT) receptors and vestibular pathways, Usman and Balaban (1) showed the distribution of 5-HT_1F_ receptors in vestibular and trigeminal ganglia, including blood vessels, in the monkey. They also discussed the relationship between the 5-HT_1F_ receptor and 5-HT_1B_ and 5-HT_1D_ receptors, using previously data from their laboratory. They logically explain possible interactions of triptans on 5-HT_1F_ receptors (triptans also activate 5-HT_1B_ and 5-HT_1D_ receptors) and also point out that serotonin may be involved in the pathophysiology of some migrainous features (e.g., tinnitus and phonophobia) in vestibular migraine.

In the central nervous system (CNS), serotonin neurons are involved in functions such as appetite, sleep, mood, thermoregulation, anxiety, aggression, learning and cognition, also inducing feelings of well-being and happiness. Serotonin is also an important vasoconstrictor, which may account for the vasomotor changes observed during a migraine attack.

Serotonin, a monoamine neurotransmitter, is encountered in significant quantities in the CNS and gastrointestinal tract. Nausea and vomiting are pathognomonic features of a migraine attack, and serotonin agonists may be a strong vomiting and nausea trigger. Furthermore, reduction in brain synthesis of serotonin by tryptophan depletion was able to provoke more intense headache, nausea, and photophobia in migraine patients (2).

In vestibular migraine, the balance disorder is often associated with nausea and vomiting, again pointing to the pivotal role of the central serotonergic system in the pathophysiology of migraine (3).

The evolutionary role of serotonin in feeding, i.e., food selection and absorption, must have occurred. Particular types of food or beverage may trigger migraine attacks (4–7). In contrast, attacks are also provoked by fasting (8). Poisonous or toxic food is eliminated by induced vomiting or diarrhea through a serotonergic mechanism. Curiously, on the phylogenetic scale, serotonin may be found in fungi and plants (9). Plant spines and some venoms secreted by insects may contain serotonin, which causes pain in the threatening animal to ward off the danger. This also shows its important role in self-defense and induction of pain, even in primitive forms of life.

Serotonin is also encountered in some seeds and fruits, helping the digestive function. Ninety percent of the serotonin of the body is located in the gastrointestinal tract (enterochromaffin cells), whose function is to regulate gastrointestinal mobility, fundamental in food absorption. There is a bidirectional communication between gut and CNS. The gut transmits information to CNS through multiple forms, including vagal and spinal neurons, and gut receives CNS outputs by autonomic neurons and neuroendocrine regulation (10).

An evolutionary role of migraine in guaranteeing survival and successful reproduction was described (11). Migraine probably exerted a role in the organization of the world’s first cities, transforming a previously nomadic existence into a sedentary way of life (12). Conditions such as incapacitating migraine attacks, pregnancy, lactation, and menses may well be states that led our species to its present-day “civilized” way of life (12).

Some individuals present a worsening in their migraine attacks when changes in the environment occur, e.g., emotional or physical stress. Regarding feeding, distinct smells, specific food ingestion, and food privation might all have triggered migraines in these migraine-prone individuals who, by changing their behavior, may have forced the entire group to reconsider their previous migratory way of life (12).

My main comment on serotonin and its relation to vomiting is that, since the time of Hippocrates, it has been known that, in some individuals suffering a migraine attack, the crisis is completely relieved after an episode of vomiting (13). Some migraineurs, on realizing that an episode of vomiting can stop a migraine attack, induce vomiting in order to curtail the attack (14–16). In some series, up to 15% of the migraineurs reported using induced vomiting in an attempt to bring to an end a migraine crisis (14–16).

The question still to be answered is: why does this occur? Interestingly, to date, no drug has been able to provide immediate relief of the migraine pain. The good therapeutic response expected with the use of analgesic and migraine-specific drugs is a progressive decrease in the pain intensity lasting up to 2 h in migraineurs. In this context, Chai and coworkers (10) recently discussed some hypotheses for how vomiting could stop migraine attacks.

Serotonin antagonists have largely been used as antiemetic drugs, particularly 5-HT_3_ receptor antagonist (17), but their effects on the pain are weak or non-existent. There is clearly a myriad of different serotonin receptor subtypes, each of which has a particular function depending on the neuronal system where it is located and on the various inputs that may influence the neuronal response. The chemoreceptor trigger zone in the *area postrema*, a brain structure that acts as a vomit-inducing center in the medulla, has different subtypes of serotonin receptors and, due to the absence of the blood–brain barrier, circulating substances in the blood or cerebrospinal fluid may either inhibit or stimulate the mechanism of vomiting.

Over the past few decades, a number of drugs that act on serotonin receptors or by regulating the serotonin concentration in synapses have been developed and introduced in clinical practice, both to treat acute headache attacks and to preventively control incapacitating crises in a migraineur.

Interestingly, serotonin-modulating drugs such as reuptake inhibitors (e.g., fluoxetine and citalopram) have no clear efficacy for migraine prevention but also do not seem to make migraine attacks any worse. The marginal beneficial effects (18–21), if any, on the migrainous features claimed by some, may also be due to control of the depressive and anxiety symptoms by these antidepressants/anxiolytics drugs, depression and anxiety being comorbidities, which might influence the frequency of migraine attacks (21, 22).

Even now, with all the information available on serotonin and its receptors, we are still far from elucidating the mechanism involved in a migraine attack. Thus, studies such as this (1) are essential for understanding the pathogenesis of migraine and planning a rational form of treatment (23). Understanding the mechanism through which vomiting may interrupt a migraine attack may also help in the development of new drugs with a more effective action in interrupting migraine attacks more swiftly (see Figure 1).

**Figure 1:**
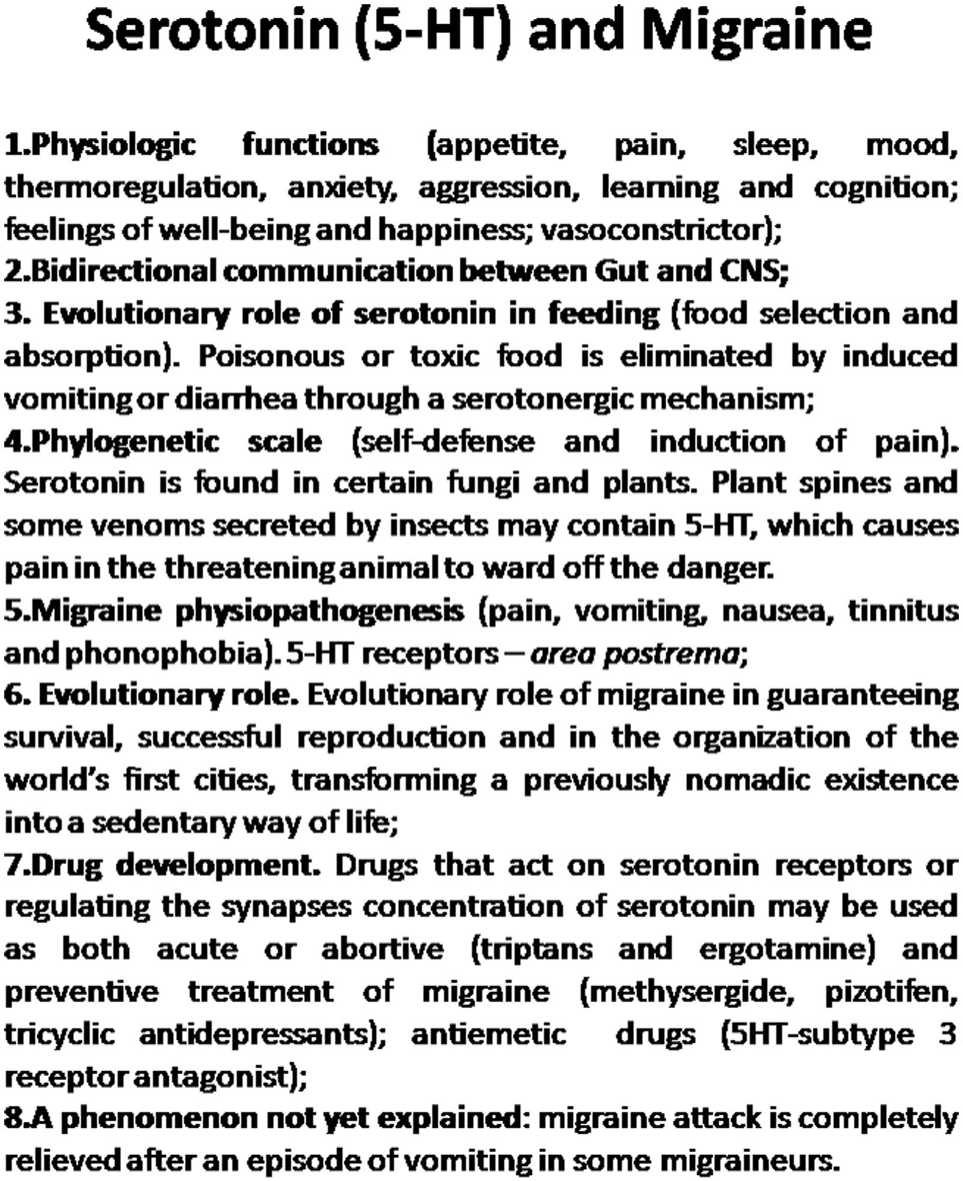
**The role of serotonin in migraine physiopathogenesis, phylogenetic scale, human evolution, and drug development**.

## Author Contributions

The author confirms being the sole contributor of this work and approved it for publication.

## Conflict of Interest Statement

The author declares that the research was conducted in the absence of any commercial or financial relationships that could be construed as a potential conflict of interest.
